# Increased Corticomuscular Coherence and Brain Activation Immediately After Short-Term Neuromuscular Electrical Stimulation

**DOI:** 10.3389/fneur.2018.00886

**Published:** 2018-10-23

**Authors:** Rui Xu, Yaoyao Wang, Kun Wang, Shufeng Zhang, Chuan He, Dong Ming

**Affiliations:** ^1^Lab of Neural Engineering & Rehabilitation, Department of Biomedical Engineering, College of Precision Instruments and Optoelectronics Engineering, Tianjin University, Tianjin, China; ^2^Tianjin International Joint Research Center for Neural Engineering, Academy of Medical Engineering and Translational Medicine, Tianjin University, Tianjin, China

**Keywords:** neuromuscular electrical stimulation, corticomuscular coherence, event-related desynchronization, functional connection, brain activation

## Abstract

Neuromuscular Electrical Stimulation (NMES) is commonly used in motor rehabilitation for stroke patients. It has been verified that NMES can improve muscle strength and activate the brain, but the studies on how NMES affects the corticomuscular connection are limited. Some studies found an increased corticomuscular coherence (CMC) after a long-term NMES. However, it is still unknown about CMC during NMES, as relatively pure EMG is very difficult to obtain with the contamination of NMES current pulses. In order to approach the condition during NMES, we designed an experiment with short-term NMES and immediately captured data within 100 s. The repetition of wrist flexion was used to realize static muscle contractions for CMC calculation and dynamic contractions for event-related desynchronization (ERD). The result of 13 healthy participants showed that maximal values (*p* = 0.0020) and areas (*p* = 0.0098) of CMC and beta ERD were significantly increased immediately after NMES. It was concluded that a short-term NMES can still reinforce corticomuscular functional connection and brain activation related to motor task. This study verified the immediate strengthen of corticomuscular changes after NMES, which was expected to be the basis of long-term neural plasticity induced by NMES.

## Introduction

Neuromuscular Electrical Stimulation (NMES) is a technique that can generate contractions of paralyzed or paretic muscles by applying electrical current on these muscles (1). Confidential evidence has shown that NMES can increase the maximal voluntary contraction and neural activation assessed by the twitch interpolation technique (2). Poststroke rehabilitation with NMES has been found to effectively prevent muscle atrophy, improve muscle strength (1, 3) and coordination (4). More recently, a study published in *Nature Communications* revealed the efficacy and mechanisms of brain-actuated functional electrical stimulation via the clinical performance and functional connectivity (5).

The influence of NMES on muscles is easy to understand as NMES is directly applied to muscles. However, the effect in muscles is not enough to realize motor rehabilitation, since the brain plays an important role in motor recovery. Phenomena of event-related desynchronization/synchronization (ERD/ERS) of EEG could be found at the frontal and parietal areas when limb movements are executed or imagined, which shows a power decrease/increase in the alpha (8–13 Hz) and beta (14–30 Hz) bands (6). The ERD pattern was used to indicate brain activation and sensitive to different movement speed on action observation (7). What's interesting is that NMES applied on muscles also affects Electroencephalogram (EEG) oscillatory (8), which verifies that NMES on the muscles can activate related brain area, and this activation pattern represented by ERD is similar to that under active movement. Lo et al. used near infrared spectroscopy (NIRS) to investigate cortical activation of different-intensity electrical stimulations (9). These studies evaluated the efficacy of NMES from the view of brain activation.

Moreover, corticomuscular coherence (CMC) is a method to estimate neural coupling via Magnetoencephalogram (MEG) or EEG and Electromyogram (EMG). CMC has drawn much attention since it was first discovered by Conway et al. (10). For now, we have known that the strength of CMC is adjusted or affected by attention (11), muscle contraction type (12, 13), muscle contraction force (14, 15), muscle fatigue (16, 17), and motor learning (18). As CMC statistically calculated the synchronization between the brain and muscle signals, it reflects functional connection between the motor cortex and muscles (19). Due to this, CMC of stroke patients has obtained some focus since Mima et al. first revealed that there was significant EEG-EMG coherence only in the unaffected side of the brain (20). Except for the amplitude of CMC, the location is still different for stroke patients and the control. Rossiter et al. found that the CMC of stroke patients were located more widely than healthy people (21). It may verify that brain regions in the contralesional hemisphere were involved to help recover motor functions. In 2017, the result of an interesting study demonstrated that although CMC was reduced in the acute phase after stroke, there was no significant change within the following 4 ~ 6 weeks despite of improved behavioral performance (22). Maybe CMC is not an efficient marker for early recovery of motor function following stroke. The continuous learning of CMC should help us make CMC more sensitive to the rehabilitation of stroke.

CMC calculation provides a new perspective to study the efficacy of NMES. Lai et al. have done interesting and important exploration on the EEG-EMG coherence affected by long-term sensory electrical stimulation (23, 24). They found that the electrical stimulation causing no muscle contraction and pain increased the EEG-EMG coherence. The accurate CMC during NMES is also necessary as it provides direct information on the effect of NMES, and reflects transient neural plasticity. However, it is difficult to obtain pure EMG, as the stimulation current contaminates EMG severely. Therefore, we designed an experiment to capture the immediate effect of a short-term NMES and analyzed both functional connection and brain activation via CMC and ERD respectively. We hypothesized that CMC could be strengthened immediately after NMES.

## Materials and methods

### Participants and experiments

Thirteen healthy right-handed people (5 females and 8 males; mean age: 21.2 ± 1.1 years old) from Tianjin University participated in the study. The participants had no history of neuromuscular disorders. The study was approved by the ethics committee of Tianjin University. All participants signed informed consent in advance.

**Figure 1:**
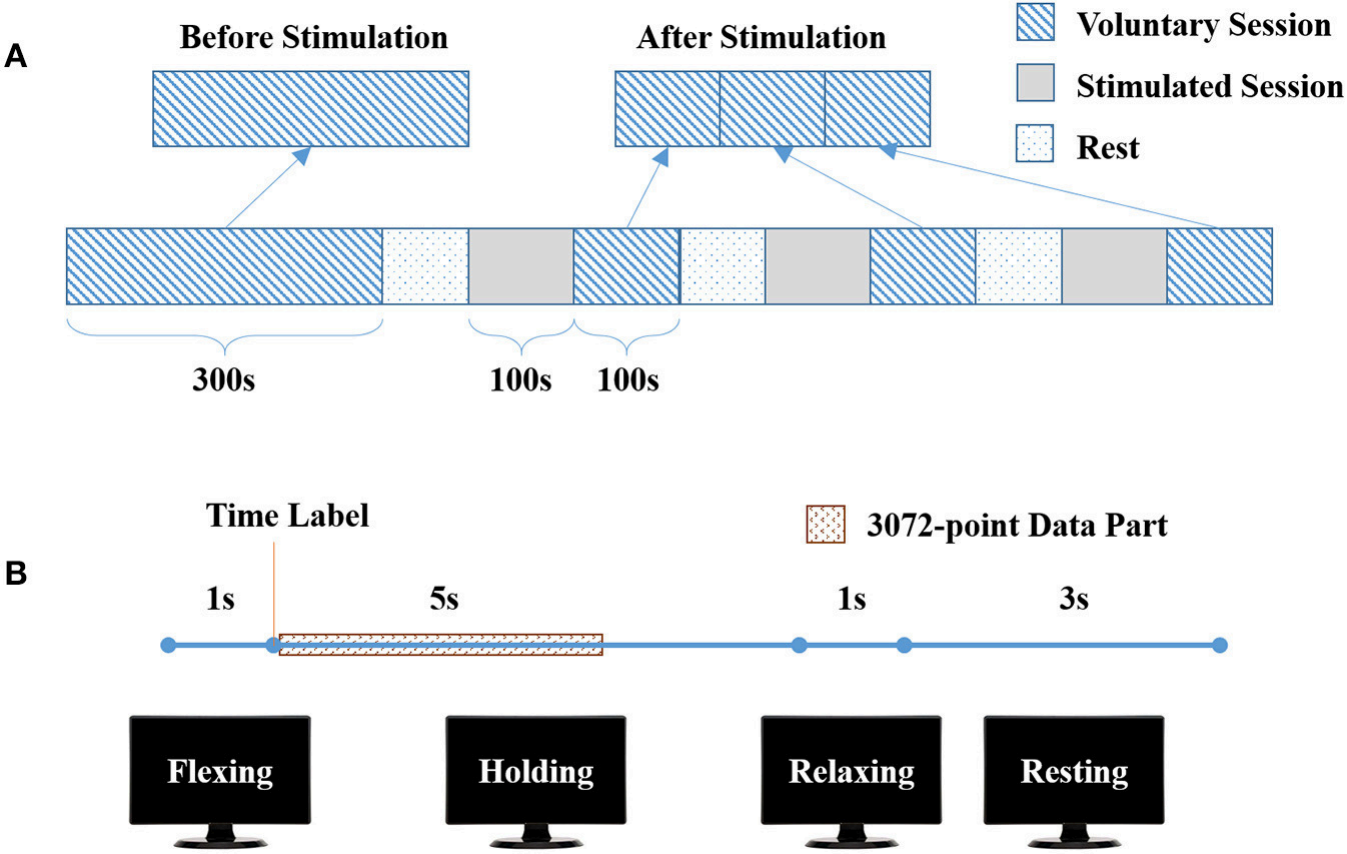
Experimental protocol. **(A)** The complete experiment, consisting of one long voluntary sessions (300 s) and three stimulated plus short voluntary sessions (100 s + 100 s). **(B)** One trial in voluntary sessions, with 1-s wrist flexing, 5-s flexion holding, 1-s wrist relaxing and 3-s wrist resting.

The experiment consisted of one long voluntary session (300 s) and three stimulated plus short voluntary sessions (100 s + 100 s) shown in Figure 1A. There was a rest for 5 to 10 min between two sessions. There were 30 trials in the long voluntary session, and 10 trials in each short voluntary session. Each voluntary trial started with 2-s wrist flexing, followed by 5-s wrist flexion holding and 1-s relaxing, and ended up with 3-s resting (Figure 1B).

**Figure 2:**
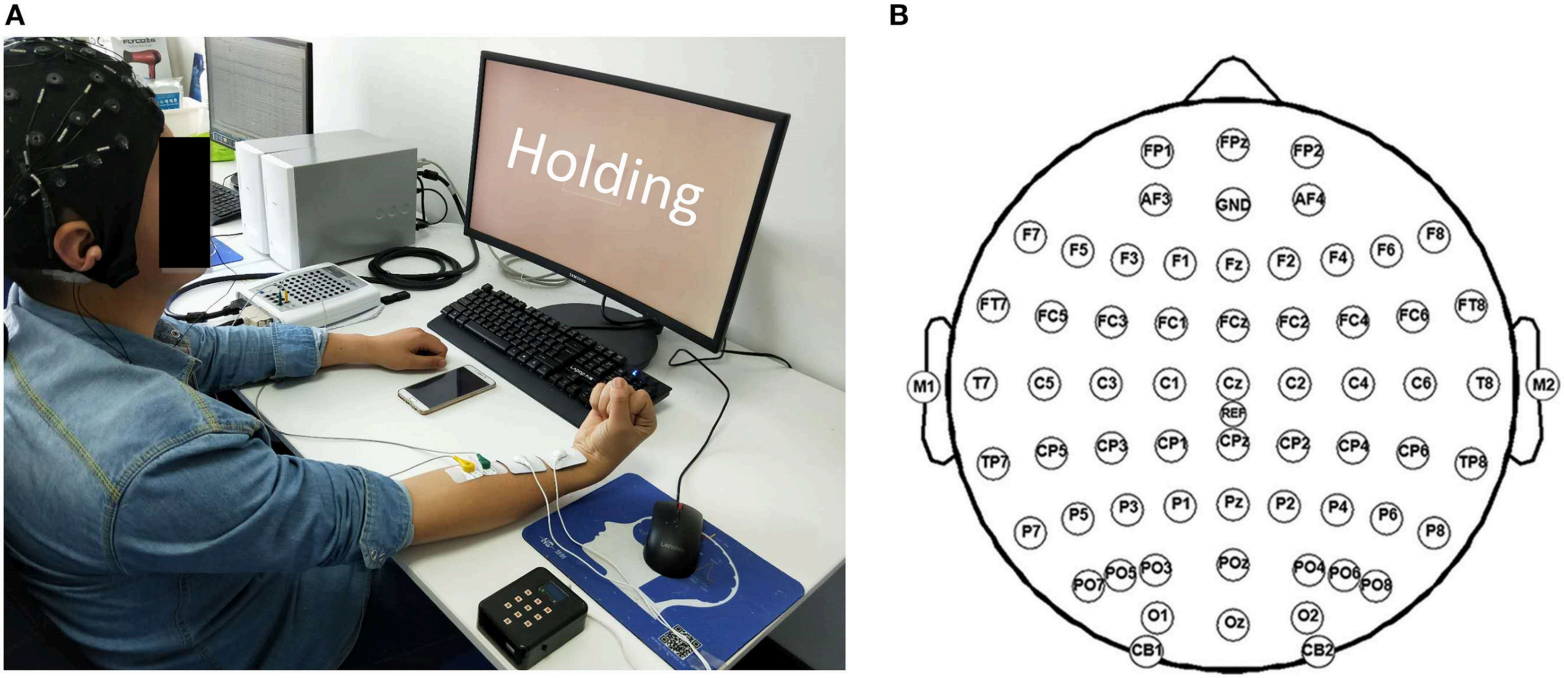
Experiment scene. **(A)** The photo of one participant during the experiment. (**B**) Channel locations according to the international 10–20 system.

Before the experiment, the participant was seated in front of a 17” monitor with his right arm on the table (Figure 2A). His right hand was relaxed to make a slight fist. During the experiment, the participant followed the instructions on the monitor (generated by Psychtoolbox within Matlab) to complete each trial: he flexed his right wrist when “Flexing” showed up in the monitor, held the flexed wrist for the “Holding” part, and then relaxed and rested according to the cue (Figure 1B). There was a time label at the onset of holding part. During the stimulated session, the participant was seated still like in the voluntary session and his/her wrist was relaxed without any voluntary movement. His/Her right flexor carpi radialis (FCR) was electrically stimulated for 100 s, with the stimulation frequency at 30 Hz and the peak current varying from 7 to 13 mA for different participants (mean: 10.9 ± 2.3 mA). The wrist of the participant was kept flexed under this stimulation. The stimulation intensity was determined at each participant's tolerance with an actual wrist flexion before the first stimulated session: the peak current was raised from 5 mA by 1 mA each time until the participant felt uncozy and asked to stop (the maximal current), and the peak current used in the stimulated session was 1 mA less than the maximal current. The maximal current is listed individually in Table 1.

**Table 1:** The peak current and C3 EEG-EMG coherence for individuals.

**No. of participant**	**Peak current (mA)**	**Max of** ***Coh**_***s***_*		**Area of** ***Coh***_*****s*****_	
		**Before**	**After**	**Before**	**After**
1	7	0.0018	**0.0235**	0.0018	**0.0337**
2	13	0.0019	**0.0143**	0.0019	**0.0162**
3	9	0.0005	**0.0115**	0.0005	**0.0188**
4	13	0.0122	**0.0131**	0.0231	0.0138
5	10	0	**0.0322**	0	**0.0389**
6	13	0.0210	**0.1029**	0.0376	**0.1542**
7	11	0.0107	**0.0148**	0.0107	**0.0227**
8	10	0.0026	**0.0069**	0.0034	**0.0095**
9	15	0	0	0	0
10	10	0.0029	**0.0068**	0.0029	**0.0122**
11	10	0.0005	0	0.0005	0
12	8	0.0008	**0.0016**	0.0008	**0.0016**
13	13	0	0	0	0

*The values in bold indicate increases after NMES*.

### Data recording and preprocessing

EEG and surface electromyography (sEMG) data were acquired simultaneously with a Neuroscan SynAmps2 amplifier, hardware-filtered in the frequency range of 0.015–250 Hz and sampled at 1,000 Hz. EEG data was recorded with 64 electrodes located in the positions following the 10/20 system (Figure 2B), while sEMG data was recorded by 2 Ag/AgCl electrodes placed on the surface of FCR (2-cm interelectrode distance). The recorded data were referenced to the nose and grounded at the prefrontal lobe. An additional 50-Hz notch filter was used during data acquisition.

Data analyses were performed using Matlab R2017b (MathWorks, MA, USA), with the toolbox EEGLAB (Swartz Center for Computational Neuroscience; http://sccn.ucsd.edu/eeglab/). The acquired EEG data at C1, C3, and C5 electrodes were re-referenced using the surface Laplacian technique (25) according to (1), (2), and (3).

(1) $$\begin{array}{l} V_{C1^{\prime }}=V_{C1}-\left(V_{FC1}+V_{CP1}+V_{C3}+V_{C\text{z}}\right)/4 \end{array}$$

(2) $$\begin{array}{l} V_{C3^{\prime }}=V_{C3}-\left(V_{FC3}+V_{CP3}+V_{C1}+V_{C5}\right)/4 \end{array}$$

(3) $$\begin{array}{l} V_{C5^{\prime }}=V_{C5}-\left(V_{FC5}+V_{CP5}+V_{C3}+V_{T7}\right)/4 \end{array}$$

where *V*_*I*_ (*I* = *C1, FC1, CP1, Cz, C3, FC3, CP3, C5, FC5, CP5*, or *T7*) indicates the EEG data acquired at the electrode *I* and $V_{C1^{\prime }}$, $V_{C3^{\prime }}$, and $V_{C5^{\prime }}$ were the re-referenced EEG data at C1, C3, and C5. Then a 4th-order zero-phase Butterworth filter was used to obtain filtered EEG data (5 ~ 45 Hz) and sEMG data (20~250 Hz). The full-wave rectified sEMG were obtained as the absolute value of the data.

There were 30 trials (in the long voluntary session) before and 10 × 3 trials (in the short voluntary sessions) after the stimulated sessions (Figure 1A). A 3072-point data part started from the time label was extracted from the “Holding” part of each trial (Figure 1B). The re-referenced C1 EEG, C3 EEG, C5 EEG, and sEMG data before or after stimulation consisted of 30 data parts. Each data part was further divided into 6 segments of 512 points. In total, 180 data segments were used to calculate the EEG-EMG coherence.

### CMC

Denoting the fast Fourier transform (FFT) of the *i*th segment of C3 EEG by *X*_*i*_(*f*) and of the *i*th segment of rectified sEMG by *Y*_*i*_(*f*), the coherence (*Coh*) at frequency *f* was estimated as:

(4) $$\begin{array}{l} Coh\left(f\right)=\frac{{\left|\overset{N}{\underset{i=1}{\sum }}X_{i}^{*}\left(f\right)Y_{i}\left(f\right)\right|}^{2}}{\overset{N}{\underset{i=1}{\sum }}X_{i}\left(f\right)X_{i}^{*}\left(f\right)\overset{N}{\underset{i=1}{\sum }}Y_{i}\left(f\right)Y_{i}^{*}\left(f\right)} \end{array}$$

where *i* = 1, …, *N* is the number of data segments available for analysis, and ^*^ denotes complex conjugate. The use of 512-point segments with a sampling rate of 1,000 Hz provided a 1.95 Hz frequency resolution in the coherence spectra. The C1 and C5 EEG-EMG coherence was calculated in the same way.

The confidence level for the coherence (26) was calculated as:

(5) $$\begin{array}{l} CL\left(\alpha \right)=1-{\left(1-\alpha \right)}^{\frac{1}{N-1}} \end{array}$$

where *N* is the number of data segments and α is the desired level of confidence. We considered coherence to be significant above the 95% confidence limit (α = 0.95). As there were 180 segments for coherence calculation, CL was 0.0166 according to (5).

The significant coherence *Coh*_*s*_ used in this study was calculated as (6). This calculation neglected the small differences of CMC below CL. The maximal value of *Coh*_*s*_ was also used to indicate the strongest corticomuscular connection before or after NMES.

(6) $$Coh_{s}\left(f\right)=\{\begin{array}{cc} Coh\left(f\right)-0.0166 & if\;Coh\left(f\right)>0.0166\\ 0 & else \end{array}$$

The mean curve of significant coherence *M*_*coh*_ was obtained by (7).

(7) $$\begin{array}{l} M_{Coh}\left(f\right)=\frac{1}{K}\overset{K}{\underset{i=1}{\sum }}Coh_{s}\left(f\right) \end{array}$$

where *K* is the total number of participants.

#### Area of significant coherence

The C1, C3, and C5 EEG-EMG coherence values below CL were set to zero according to (6). Only the significant coherence was used in the area calculation. The area of significant coherence (*A*_*Coh*_) within 5 ~ 45Hz can be used to estimate the strength of corticomuscular coupling (18), and it was calculated as:

(8) $$\begin{array}{l} A_{Coh}=\overset{45Hz}{\underset{f=5Hz}{\sum }}Coh_{s}\left(f\right) \end{array}$$

#### Center of gravity for frequency

To detect the frequency shifts of the coherence spectrum, we calculated the Center of Gravity for the frequency (*CoG*_*f*_), that is, the frequency at which coherence is concentrated and balanced. The *CoG*_*f*_ of C3 EEG-EMG coherence was obtained by (9).

(9) $$\begin{array}{l} CoG_{f}=\frac{\overset{n}{\underset{i=1}{\sum }}f_{i}\cdot Coh_{s}\left(f_{i}\right)}{\overset{n}{\underset{i=1}{\sum }}Coh_{s}\left(f_{i}\right)} \end{array}$$

where *i* = 1,…,*n* indicates the number of significant bins with its respective frequency *f*_*i*_ and coherence *Coh*_*s*_.

#### Median frequency of sEMG

In order to exclude the effect of muscle fatigue on CMC, we also calculated the median frequency of sEMG before and after the stimulated sessions. The median frequency is defined as the frequency that divided the spectrum into two equal areas. It has been widely used in the studies related to muscle fatigue (27, 28). The median frequency of sEMG during different sessions were calculated and compared to indicate the fatigue states in this study.

### ERD

The re-referenced and filtered EEG data at C3 channel was downsampled to 200 Hz for ERD analysis. The event-related spectral perturbation (ERSP) method allowed us to inspect the spectral power changes of EEG in the view of time-frequency domain. Therefore, ERSP was calculated as:

(10) $$\begin{array}{l} ERSP\left(f,t\right)=\frac{1}{n}\overset{n}{\underset{i=1}{\sum }}\left(F_{i}{\left(f,t\right)}^{2}\right) \end{array}$$

where *i* = 1, …, *n* is the number of trials, and *F*_*i*_(*f, t*) is the spectral estimation of the *i*th trial at frequency *f* and time *t* (29). Short-time Fourier transform (STFT) was used to perform time-frequency analysis with a Hanning window. The number of windows was set to 200 with the length of 512 points. ERSP was calculated using 30 trials of data and the data length was 10 s for each trial, with 2 s before movement onset and 8 s after (1-s flexing, 5-s holding, 1-s relaxing and 1-s resting). Baseline-normalized ERSP was calculated relative to the baseline period (before movement onset).

In order to investigate the difference of brain oscillation before and after NMES, the ERD at C3 within 1 s after the movement onset was extracted as follows:

(11) $$\begin{array}{l} ERD\left(f\right)=\overset{1s}{\underset{t=0s}{\sum }}ERSP\left(f,t\right) \end{array}$$

The first second after movement onset indicated the wrist flexion period, excluding the holding part.

### Statistical analysis

All the features mentioned above before and after NMES, including the maximal values and areas of *Coh*_*s*_, *CoG*_*f*_, and ERD values, were compared with Wilcoxon signed rank test. The significance was calculated two-tailed. For the *CoG*_*f*_, only data with significant coherence both before and after NMES were considered.

## Results

### CMC

**Figure 3:**
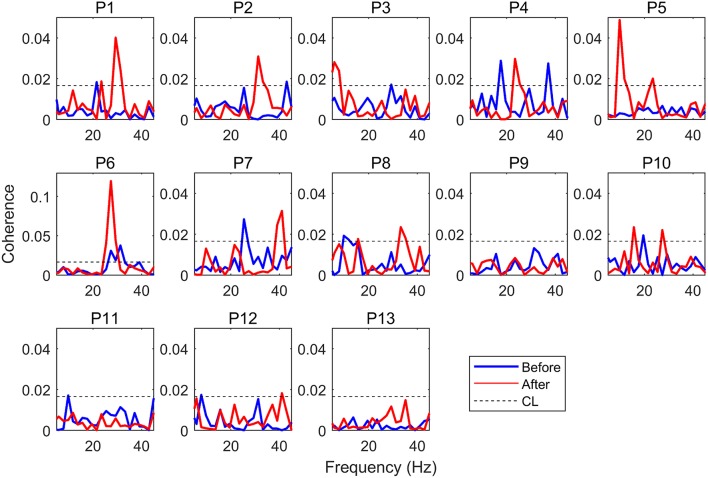
EEG-EMG coherence. P1 to P13 indicate Participant 1 to Participant 13, the blue line indicates coherence before NMES, while the red line indicates coherence after NMES.

We calculated the EEG-EMG coherence of all the subjects. The C3 EEG-EMG coherence before (blue line) and after (red line) NMES of each participant is listed in Figure 3. There were some participants who did not present significant EEG-EMG coherence before or after NMES, such as P9, P11, P12, and P13.

**Figure 4:**
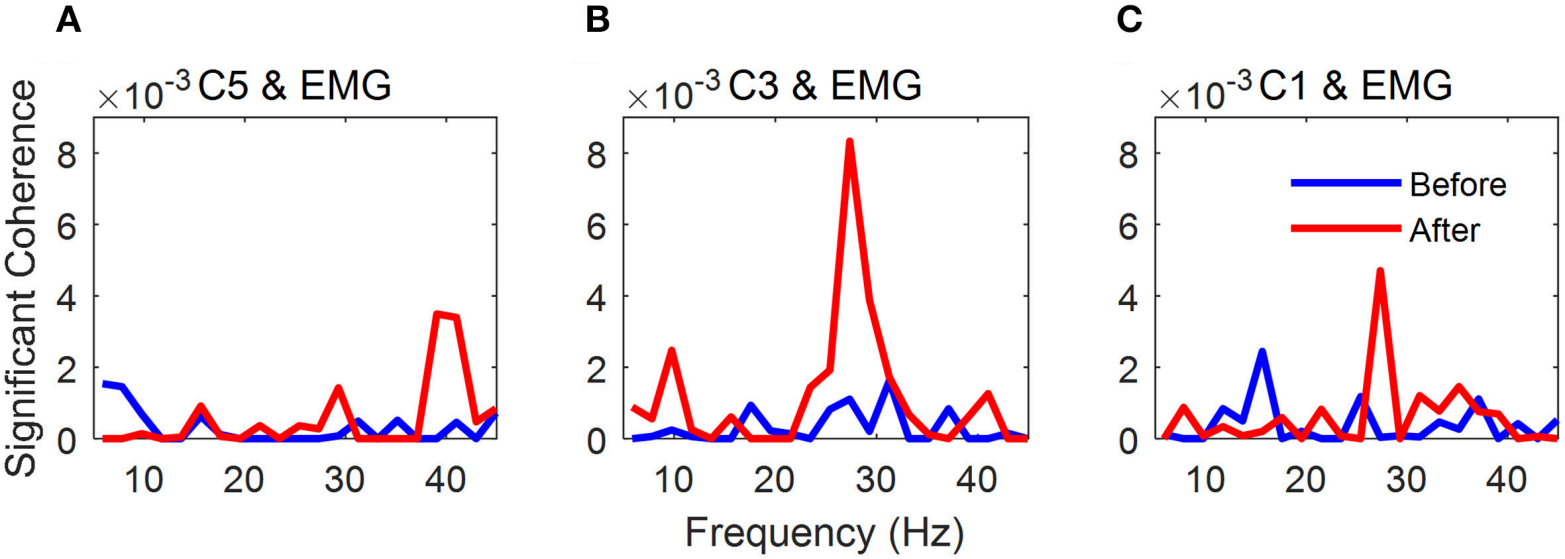
Grand average of significant EEG-EMG coherence. **(A)** C5 EEG-EMG coherence. **(B)** C3 EEG-EMG coherence. **(C)** C1 EEG-EMG coherence. The blue line indicates significant coherence before NMES, while the red line indicates significant coherence after NMES.

The grand average of significant coherence is shown in Figure 4. The peak values of mean coherence after NMES were larger than those before NMES in Figures 4A–C. It was obvious that NMES had different influence on these three channels of coherence. The maximal values and areas of significant coherence (listed in Table 1) were calculated for further statistical analysis. The Wilcoxon signed rank test was used and the result indicated in Table 2 that the maximal value and area of significant coherence for C3 EEG-EMG coherence after NMES was significantly larger than those before NMES (Max: *p* = 0.0020; Area: *p* = 0.0098). Although areas of C1 and C5 EEG-EMG coherence after NMES also increased, there was no significant difference.

**Table 2:** Maximal value and area of significant coherence.

	**Max of** ***Coh***_*****s*****_		**Area of** ***Coh***_*****s*****_	
	**Before**	**After**	**Before**	**After**
C5 EEG & EMG	0.0050 ± 0.0073	0.0058 ± 0.0123	0.0067 ± 0.0097	0.0118 ± 0.0329
C3 EEG & EMG	0.0042 ± 0.0064	**0.0175** ±0.0274^*^	0.0064 ± 0.0114	**0.0247** ±0.0408^*^
C1 EEG & EMG	0.0056 ± 0.0087	0.0101 ± 0.0162	0.0082 ± 0.0135	0.0127 ± 0.0188

The values in bold are with significant difference compared to the values before NMES.

* *p < 0.05*.

**Figure 5:**
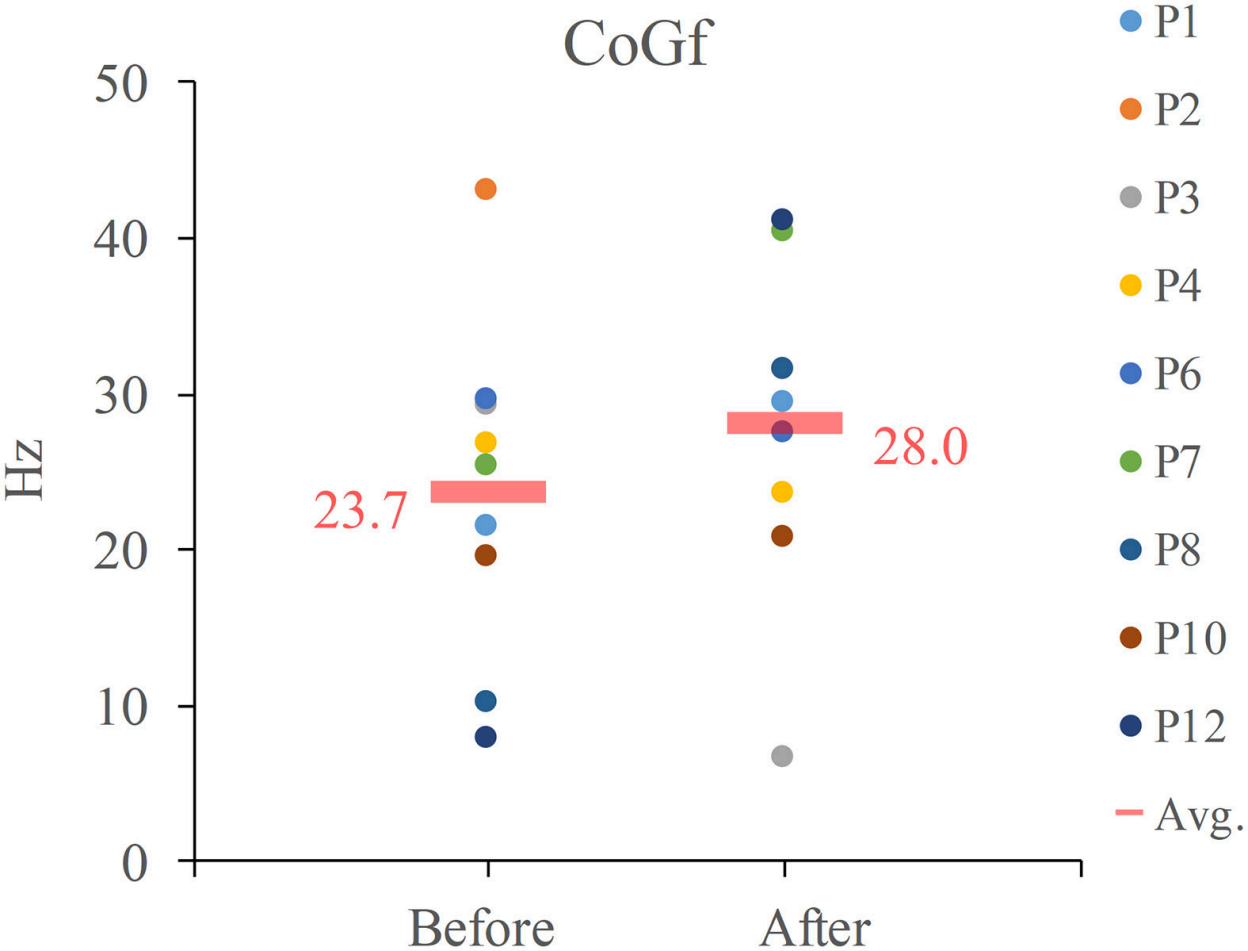
*CoG*_*f*_ for EEG-EMG coherence. Avg. indicates average.

There were only 9 participants who showed significant C3 EEG-EMG coherence both before and after NMES. The *CoG*_*f*_ of these 9 participants, its average and median values were listed in Figure 5. The average frequency was increased after NMES (Avg.: from 23.7 to 27.8 Hz), but there was no significant frequency shift after NMES according to the result we obtained.

**Figure 6:**
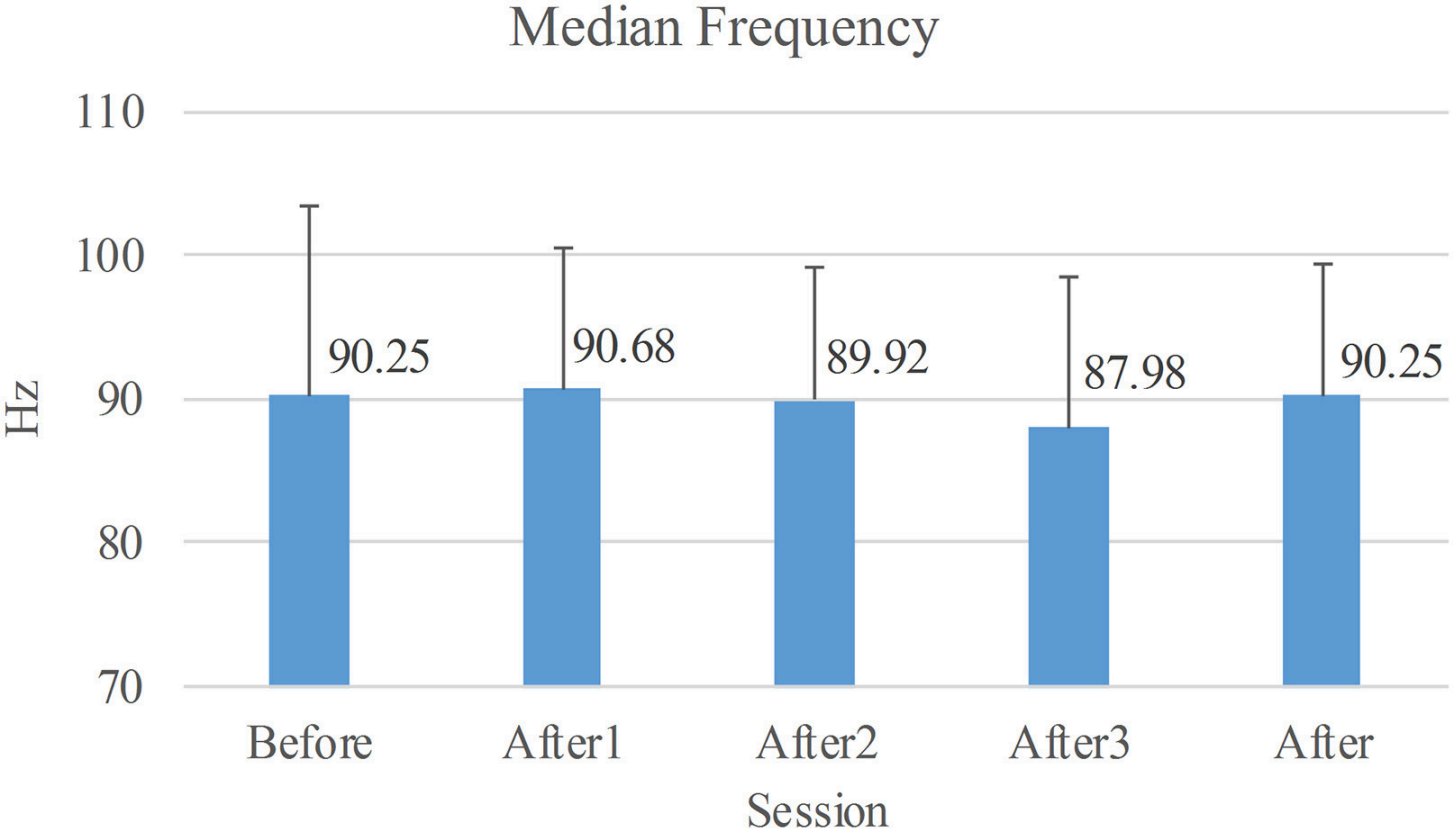
Average of median frequencies of sEMG. Before: the long voluntary session before NMES sessions. After1/2/3: the short voluntary session after the 1st/2nd/3rd stimulated session. After: the voluntary session composed of the three short voluntary sessions after the stimulated sessions.

The median frequencies of sEMG are shown in Figure 6. The one-way repeated measure ANOVA was used to compare these median frequencies, and no significant difference indicated that the fatigue state of the muscle remained the same given the sensitivity of the median frequency.

### ERD

**Figure 7:**
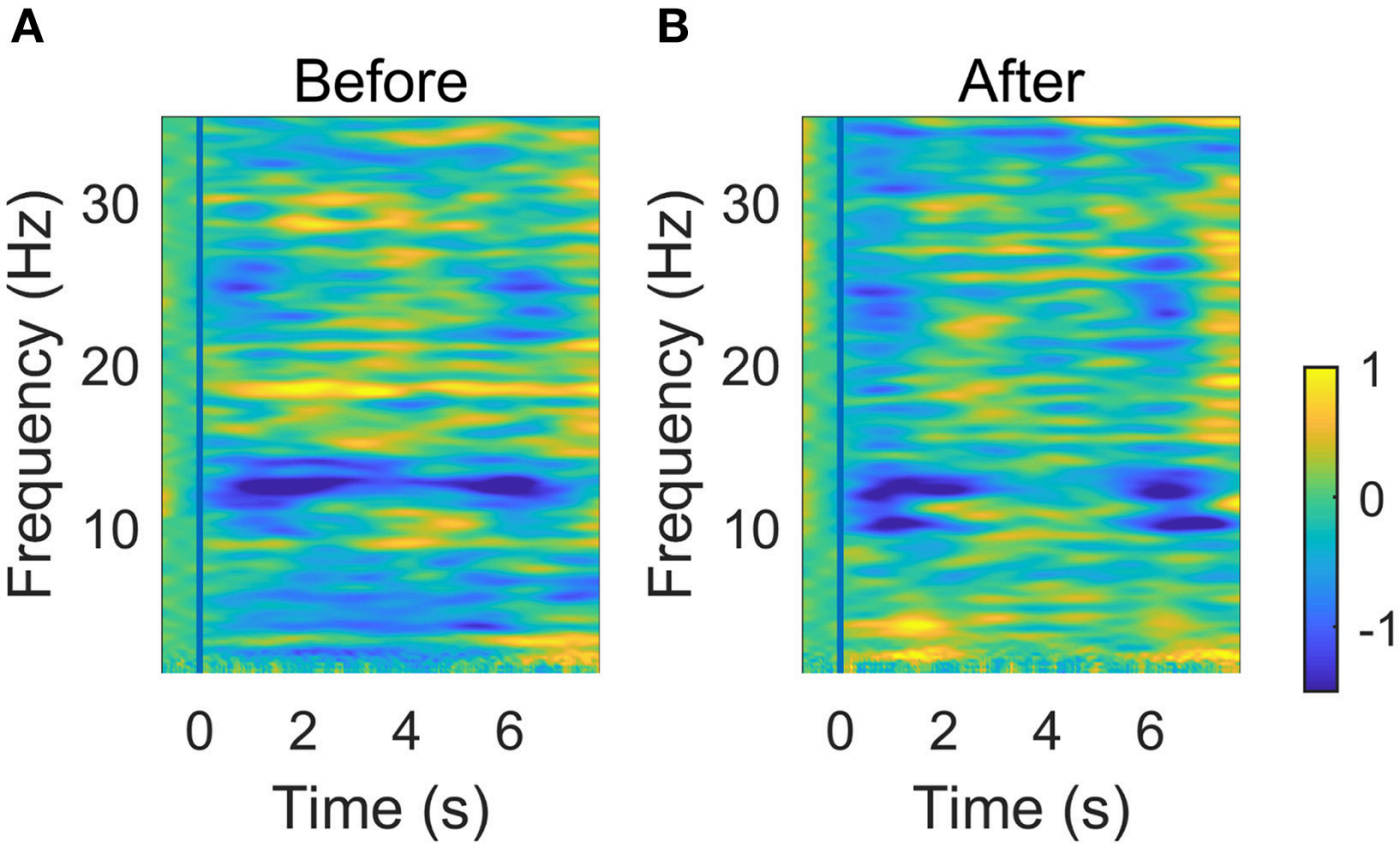
Averaged time-frequency ERSP at C3 before (**A**) and after (**B**) NMES. The vertical blue line indicates the onset of the wrist flexion.

Figure 7 shows the averaged ERSP of C3 EEG before and after NMES. There were obvious ERD patterns (blue area in the figure) in both mu (8~13 Hz) and beta (14~30 Hz) rhythms at the beginning and end of the movement part. The average ERD of certain areas are listed in Table 3. It shows that the ERD patterns seems to be weakened in the “holding” part between 1 and 6 s and the strongest ERD patterns occur mainly in mu rhythms. However, these changes are not significant according to the result of a two-way (time: Flexing, Holding, and Relaxing; frequency: mu and beta rhythms) repeated measure ANOVA.

**Table 3:** Average of ERSP.

		**Flexing (0~1s)**	**Holding (1~6s)**	**Relaxing (6~7s)**
Before	Mu Rhythms	−0.5288 ± 1.2947	−0.4502 ± 1.1315	−0.5898 ± 1.6852
	Beta Rhythms	−0.2408 ± 0.4363	−0.0512 ± 0.3655	−0.2084 ± 0.4829
After	Mu Rhythms	−0.6906 ± 1.5691	−0.3181 ± 1.4277	−0.8435 ± 2.5954
	Beta Rhythms	−0.4706 ± 0.6950	−0.1424 ± 0.4601	−0.2812 ± 1.0246

**Figure 8:**
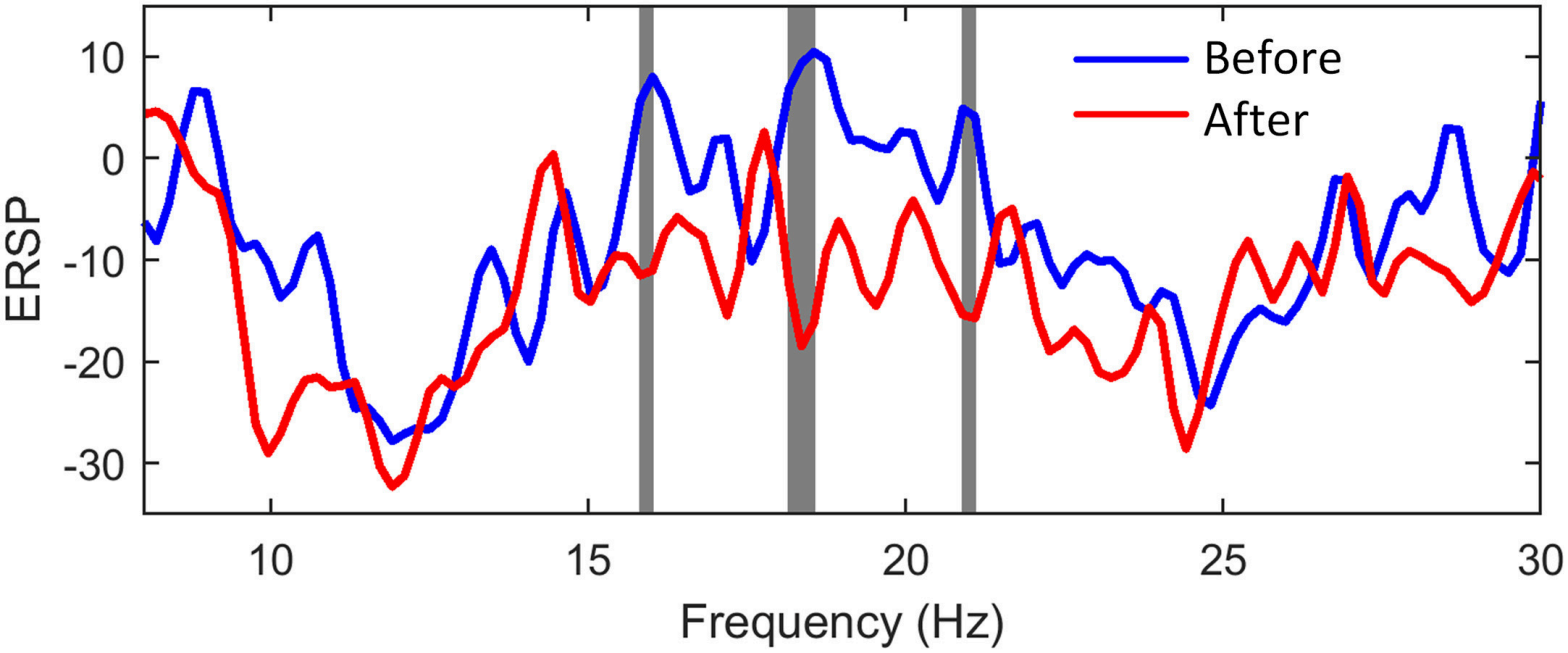
The comparison of power changes of C3 EEG. The blue line indicates ERSP before NMES, while the red line indicates ERSP after NMES. The gray blocks present statistic significant differences (*p* < 0.05) between ERSP before and after NMES.

In order to investigate and compare the brain activation before and after NMES, we calculated the ERD values of C3 EEG at different frequency bins according to (5). The Wilcoxon signed rank test was used, and the significant differences between two conditions were shaded by gray blocks in Figure 8. The blue and red lines represented normalized ERSP before and after NMES respectively. The ERD patterns were significantly stronger in three sub-beta frequency bands.

## Discussion

This study for the first time compared the coticomuscular coherence before and immediately after short-term motorial-level NMES. We designed an experiment especially for exploring the effect of NMES on the functional connectivity between the brain and muscles. It was clear that the coticomuscular coherence during active movement after NMES was significantly stronger than that before NMES. The result illustrated that NMES strengthened the interaction between the brain and muscles. We also calculated the ERD patterns before and after NMES. The analysis indicated that the ERD patterns were strengthened after NMES. It seems that NMES has a positive influence on the interaction between the brain and muscles and the activation of the brain.

There are many studies working on the rehabilitation effect of NMES. Most of them focus on the comparison of features after NMES training. For example, Sota et al. compared some gait parameters, such as the time of 10-m walking and range of motion for ankle joint, pre- and postintervention to investigate the characteristics of NMES responders (30). CMC combined with clinical functional test was used to estimate the effect of sensory NMES with motor training (24). Although the effect remained after NMES is very important, the instantaneous body response during NMES is also a key point of NMES studies. However, as the stimulation pulses affected and contaminated EMG severely, the studies on the effect during NMES are limited to comparing the features free of EMG, such as walking speed (31), ERD (8) and steady-state somatosensory evoked potential (SSSEP) (32) of the brain, and muscle thickness (33). These studies analyzed the effect of NMES on the whole body, or the brain and muscles separately, without considering motor control based on the interaction between the brain and muscles.

The mechanism of motor control can be revealed by CMC. The application of NMES was certain to cause extreme contamination of sEMG, so we had to compare the CMC before and after NMES to guarantee the data quality and result reliability. There was no studies on the effect-remaining time of NMES, but we believed that the effect of NMES should be the strongest immediately after NMES except for the effect during NMES. In this case, the stimulated session and the voluntary session after stimulation was divided into three equal parts individually. We tried to use this paradigm to approach the condition during NMES.

Although CMC has been widely studied and used (34, 35), its generation is still under debate. From the perspective of coherence, a significant coherence between two subsystems can be achieved by either one-way information flow, reciprocal communication, or the third rhythm generator affecting both (36). The result of our study verified that there were at least two directions of information flow: one was from the brain to muscles, sending cortical motor command; and the other one was from muscles to the brain, caused by NMES. As SSSEP was observed during NMES (32), the regulation of brain activities by NMES was determined. It was possible that the significantly increased CMC was the residual effect of SSSEP.

An interesting detail found in this study was that NMES increased C1, C3, and C5 EEG-EMG coherence average, but only the change in C3 EEG-EMG coherence was significant. It was deduced that the variation of coherence caused by NMES could be used to locate the cortex area in charge of the executed movement. However, whether this change varied with the location of NMES or the contracted muscles was unclear in the present study.

Higher CMC often indicated better communication between the brain and muscles, and higher beta band CMC indicate good motor performance (37). Moreover, beta-band CMC was deemed to be related to motor tasks and performance (12, 14, 15, 38). Our main result based on Table 2 was that NMES increased C3 EEG-EMG coherence, which was consistent with the newly published work of Pan et al. (24). They reported an increase of CMC after 4-weeks sensory electrical stimulation. Neural plasticity was believed to contribute to CMC increase of stroke patients, and it was crucial for learning new motor tasks (39). For healthy participants in our study, they did not learn new movement, but learn new muscle contraction patterns (NMES). This should also be regarded as learning a new motor task. According to Hebbian and homeostatic plasticity (40, 41), the CMC change after a short-term NMES reflected a transient plasticity and it could go back to former state without repetition of stimulation.

CMC could be affected by many factors. In this study, muscle contraction type and muscle force were not considered because the tasks in voluntary sessions were the same. The wrist flexed according to the clues on the screen and the data processed were extracted during the “Holding” time. The muscle contraction type in this study was static contraction, and the muscle forces in all the voluntary sessions, which was to keep the wrist flexed, should be the same. No precision requirement guaranteed that there was no difference in the attention (11). The median frequency of sEMG was analyzed to indicate no significant fatigue states. Therefore, the muscle contraction type, muscle force, attention and muscle fatigue were excluded.

The significant CMC is not a universal phenomenon for every person(18, 24). In our study, there were 4 participants who did not show significant CMC before or after NMES. Their peak currents of NMES (10, 15, 10, and 13mA) were relatively larger (the median value of these currents is 10mA). A larger stimulation current meant this participant was less sensitive to the stimulation, and he needed stronger stimulation to generate muscle contraction. Therefore, we deduced that the participant with insignificant CMC was most likely insensitive to NMES. However, this should be further verified with specifically designed experiments.

ERD patterns was often used to indicate the brain activation. In Figure 7, the weakening of ERD occurred for the holding part. This phenomenon was also shown in (42). This implied that the maintenance of the current sensorimotor state was related to the ERD rebound. The reasons may be that holding a posture was easier to execute than dynamic motor tasks, and the brain completed the static motor task in a low activation level. In order to obtain an obvious ERD variation, we compared ERD of data within the first second after the movement onset. It was found that NMES could induce a stronger ERD pattern. Vidaurre et al. found a stronger ERD pattern during NMES than motor imagery (MI), and successfully used NMES-induced patterns to decode MI (43). In our study, ERD was also strengthened in beta band after NMES. The beta ERD was linked more closely to the primary motor cortex (44, 45). Therefore, the significant cortical beta rhythm suppression showed brain activation related to motor control.

NMES can increase the excitability of human corticospinal (CS) pathways to muscles, which is usually estimated by the motor evoked potentials (MEPs). Mang et al. compared the MEPs induced by transcranial magnetic stimulation (TMS) before and after an NMES session and found that the MEP amplitude after NMES was significantly larger than that before NMES (46, 47). Whether the increase in cortical excitability is due to changes at the spinal level, cortical reorganization, or both is unclear. Such increases can strengthen CS pathways damaged by injury or disease and result in enduring improvements in function (1, 48). Here, we hypothesized that the higher CMC and stronger ERD were caused by the strengthened CS pathways.

Our study did not consider the effect caused by stimulation intensities. A study of healthy participants found that higher NMES current intensities led to greater sensorimotor network activation, and this may be attributable to increased attentional/pain processing and to increased sensorimotor integration (49). Therefore, a maximal tolerated intensity was used in our study in order to obtain significant changes of CMC before and after NMES. However, it was still unclear that how the stimulation intensity influenced CMC or whether there was a difference in CMC for sensory- and motorial-level NMES. The comparison will help understand the function of the sensorimotor circuit. The experiment was designed to approach the condition during NMES, but how CMC changed during NMES still needed to be verified.

The study was undertaken among healthy participants. Therefore, whether NMES would have the same effect in stroke patients needs to be studied further. However, NMES has been used to strengthen CS pathways in stroke rehabilitation (1, 48). It is hypothesized that the strengthened CS pathways will induce a stronger CMC in stroke patients. To be noted, the EEG channels of stroke patients for CMC calculation should be different from that of the healthy, as the lesion may be located at C3 or C4. In order to explore the effect of NMES on patients' CMC, some stroke patients will be recruited for the future work.

## Conclusion

As the estimation of NMES real-time efficacy was limited to brain or bodies separately, we designed an experiment with NMES and repeated voluntary wrist flexion, to explore the instantaneous effect after NMES. The result showed a significant increase of EEG-EMG coherence caused by NMES. Additionally, the significant increment was located in C3 position. The strengthened beta ERD indicated stronger brain activation related to motor function after NMES. Therefore, NMES not only strengthened brain activation, but it also induced a stronger connection between the brain and muscles. This result will help understand NMES-induced corticomuscular connection, and predict the body change during NMES. Based on transient neural plasticity, the immediate change after NMES lays a basis of long-term neural rehabilitation.

## Author contributions

All authors contributed to conception and design of the study and were involved in drafting and critically revising the manuscript. Additionally, RX carried out data processing and feature comparison, interpreted the results and prepared the first draft paper. YW completed EEG and EMG pre-processing. KW helped calculate ERD patterns. YW, SZ, and CH carried out the experiment. DM provided interpretation of the results and worked up the draft paper into the final version. All authors gave final approval for publication.

### Conflict of interest statement

The authors declare that the research was conducted in the absence of any commercial or financial relationships that could be construed as a potential conflict of interest.
